# A multicentre, randomised, non-inferiority clinical trial comparing a nifurtimox-eflornithine combination to standard eflornithine monotherapy for late stage *Trypanosoma brucei gambiense* human African trypanosomiasis in Uganda

**DOI:** 10.1186/s13071-018-2634-x

**Published:** 2018-02-22

**Authors:** Freddie Kansiime, Seraphine Adibaku, Charles Wamboga, Franklin Idi, Charles Drago Kato, Lawrence Yamuah, Michel Vaillant, Deborah Kioy, Piero Olliaro, Enock Matovu

**Affiliations:** 1 Busitema University Faculty of Health Sciences, Box 1460, Mbale, Uganda; 2 Moyo District Health Office, P.O. Box 1, Moyo, Uganda; 3 Ministry of Health, Box 7272, Kampala, Uganda; 4 Moyo Hospital, P.O. Box 1, Moyo, Uganda; 5 College of Veterinary Medicine, Animal Resources & Bio-security, Makerere University, P.O. Box 7062, Kampala, Uganda; 6 Armauer Hansen Research Institute, Box 1005, Addis Ababa, Ethiopia; 7 Competence Centre in Methodology and Statistics, Luxembourg Institute of Health, 1ab Rue Edison, Strassen, L-1445 Grand Duchy of Luxembourg; 8 Geneva Foundation for Medical Education and Research, 150, route de Ferney, 1211 Geneva 2, Switzerland; 9 UNICEF/UNDP/World Bank/WHO Special Programme for Research & Training in Tropical Diseases (TDR), Geneva, Switzerland

**Keywords:** Human African trypanosomiasis (HAT), Meningo-encephalitic stage, Second-stage HAT, Nifurtimox-eflornithine combination treatment (NECT)

## Abstract

**Background:**

While the combination of nifurtimox and eflornithine (NECT) is currently recommended for the treatment of the late stage human African trypansomiasis (HAT), single-agent eflornithine was still the treatment of choice when this trial commenced. This study intended to provide supportive evidence to complement previous trials.

**Methods:**

A multi-centre randomised, open-label, non-inferiority trial was carried out in the *Trypanosoma brucei gambiense* endemic districts of North-Western Uganda to compare the efficacy and safety of NECT (200 mg/kg eflornithine infusions every 12 h for 7 days and 8 hourly oral nifurtimox at 5 mg/kg for 10 days) to the standard eflornithine regimen (6 hourly at 100 mg/kg for 14 days). The primary endpoint was the cure rate, determined as the proportion of patients alive and without laboratory signs of infection at 18 months post-treatment, with no demonstrated trypanosomes in the cerebrospinal fluid (CSF), blood or lymph node aspirates, and CSF white blood cell count < 20 /μl. The non-inferiority margin was set at 10%.

**Results:**

One hundred and nine patients were enrolled; all contributed to the intent-to-treat (ITT), modified intent-to-treat (mITT) and safety populations, while 105 constituted the per-protocol population (PP). The cure rate was 90.9% for NECT and 88.9% for eflornithine in the ITT and mITT populations; the same was 90.6 and 88.5%, respectively in the PP population. Non-inferiority was demonstrated for NECT in all populations: differences in cure rates were 0.02 (95% CI: -0.07–0.11) and 0.02 (95% CI: -0.08–0.12) respectively. Two patients died while on treatment (1 in each arm), and 3 more during follow-up in the NECT arm. No difference was found between the two arms for the secondary efficacy and safety parameters. A meta-analysis involving several studies demonstrated non-inferiority of NECT to eflornithine monotherapy.

**Conclusions:**

These results confirm findings of earlier trials and support implementation of NECT as first-line treatment for late stage *T. b. gambiense* HAT. The overall risk difference for cure between NECT and eflornithine between this and two previous randomised controlled trials is 0.03 (95% CI: -0.02–0.08). The NECT regimen is simpler, safer, shorter and less expensive than single-agent DFMO.

**Trial registration:**

ISRCTN ISRCTN03148609 (registered 18 April 2008).

**Electronic supplementary material:**

The online version of this article (10.1186/s13071-018-2634-x) contains supplementary material, which is available to authorized users.

## Background

Human African trypanosomiasis (HAT) remains an important public health problem in tsetse fly infested endemic zones of rural sub-Saharan Africa. Past epidemics were fuelled by civil unrest and political upheavals, which typically disrupted health operations leading to re-appearance of historical foci [1]. It was previously reported that national control programmes cover only a proportion of the population living in endemic regions, implying gross under-estimation of prevalence. More recent estimates put 70 million people across sub-Saharan Africa at risk [2] although the incidence continues to decline, thanks to concerted efforts by the World Health Organization (WHO), several non-governmental organizations (NGOs) and national control programs.

*Trypanosoma brucei gambiense* HAT manifests in two stages: the early haemolymphatic phase is effectively treated with pentamidine [3], rendering early diagnosis essential for effective control and compliance to this relatively safe treatment regimen. Noteworthy is that pentamidin is only used for early stage *T. b. gambiense* HAT. The late or meningo-encephalitic stage of the disease, when the parasites invade the central nervous system, is invariably fatal in absence of intervention and more difficult to treat.

There have been two major advances in the treatment of late stage *T. b. gambiense* HAT. The first was the availability of eflornithine (DFMO), replacing melarsoprol, which caused life-threatening side-effects and had become increasingly ineffective [4–8]. On the other hand, unlimited access to the life-saving drug eflornithine was threatened by its complicated application (4 daily infusions over 14 days), which puts a very high nursing demand on already understaffed rural clinics where HAT is typically prevalent. Besides, eflornithine monotherapy could not have been be a long-term solution because of its trypanostatic mode of action whose success requires a competent immune system to clear the arrested parasite population [9]; resistance was presumably bound to emerge faster than usual. The second major advance was combining parenteral eflornithine with oral nifurtimox, a drug used against *T. cruzi* American trypanosoniasis. An initial pilot Bi-Therapy Trial (BTT) conducted in Omugo (north-western Uganda) in 2001 [10] was followed by a large-scale nifurtimox-eflornithine combination treatment (NECT) trials conducted in the Republic of Congo and the Democratic Republic of Congo [11]. This led to its inclusion on the WHO essential drug list [12] and to-date NECT is only used for treatment of late stage *T. b. gambiense* HAT. We undertook this non-inferiority randomised trial of NECT *versus* eflornithine in *T. b. gambiense* endemic foci of north-western Uganda, in order to corroborate findings from trials elsewhere supporting the policy change from eflornithine monotherapy to NECT.

## Methods

### Study population and sites

**Fig. 1:**
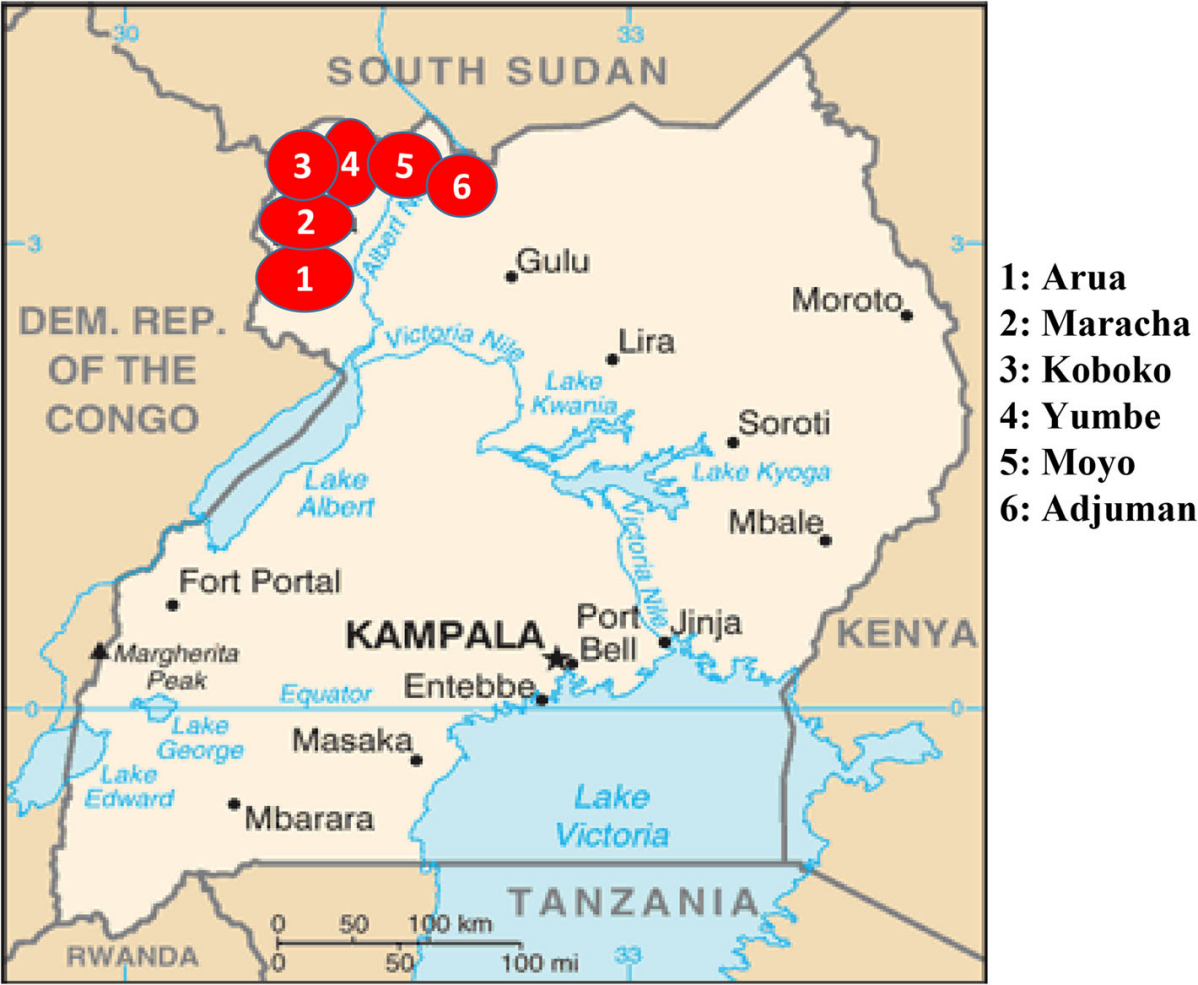
Map of Uganda showing the sites from which participants were recruited. Modified from the Central Intelligence Agency Internet resources, 2012 (https://www.cia.gov/library/publications/resources/cia-maps-publications/map-downloads/uganda-transport.jpg/image.jpg)

The study was conducted at two sites in northern Uganda, one in Omugo Health Centre IV in Arua District and the other in Moyo Hospital in Moyo District. Participants were recruited within a radius of 75 km for the Moyo site or 60 km for the Omugo site, distances that were considered feasible for follow-up. The participants came from the Arua, Maracha, Koboko and Yumbe Districts for the Omugo study arm and the Moyo and Adjumani districts for the Moyo study arm (Fig. 1).

The study population consisted of patients presenting themselves to the study site and diagnosed as having second-stage HAT (passive screening), as well as those identified as having second stage HAT during active screening campaigns conducted independently of this study or for the purpose of identifying patients for this study. We adopted a methodology aligned with previous clinical trials on second-stage trypanosomiasis [10, 11, 13–16] so as to make it possible to compare results with those from other sites. Enrolment started in November 2005 and ended in December 2007; the last patient completed follow-up in June 2009. Recruitment stopped because no eligible patients could be identified within the two catchment areas despite intense active surveillance.

### Sample size calculation

Assuming a 93% cure rate with the standard eflornithine treatment, a 10% maximum difference in cure rates between the 2 arms, a similar dropout rate, an alpha error of 5% (one-sided test) and a power of 90%, a sample size of 112 patients for each treatment arm was estimated. Allowing for a 20% dropout rate, this was adjusted to 139 patients for each treatment arm. This was rounded up to a total of 280 for the 2 arms, with an allocation ratio of 1:1.

### Study design

**Fig. 2:**
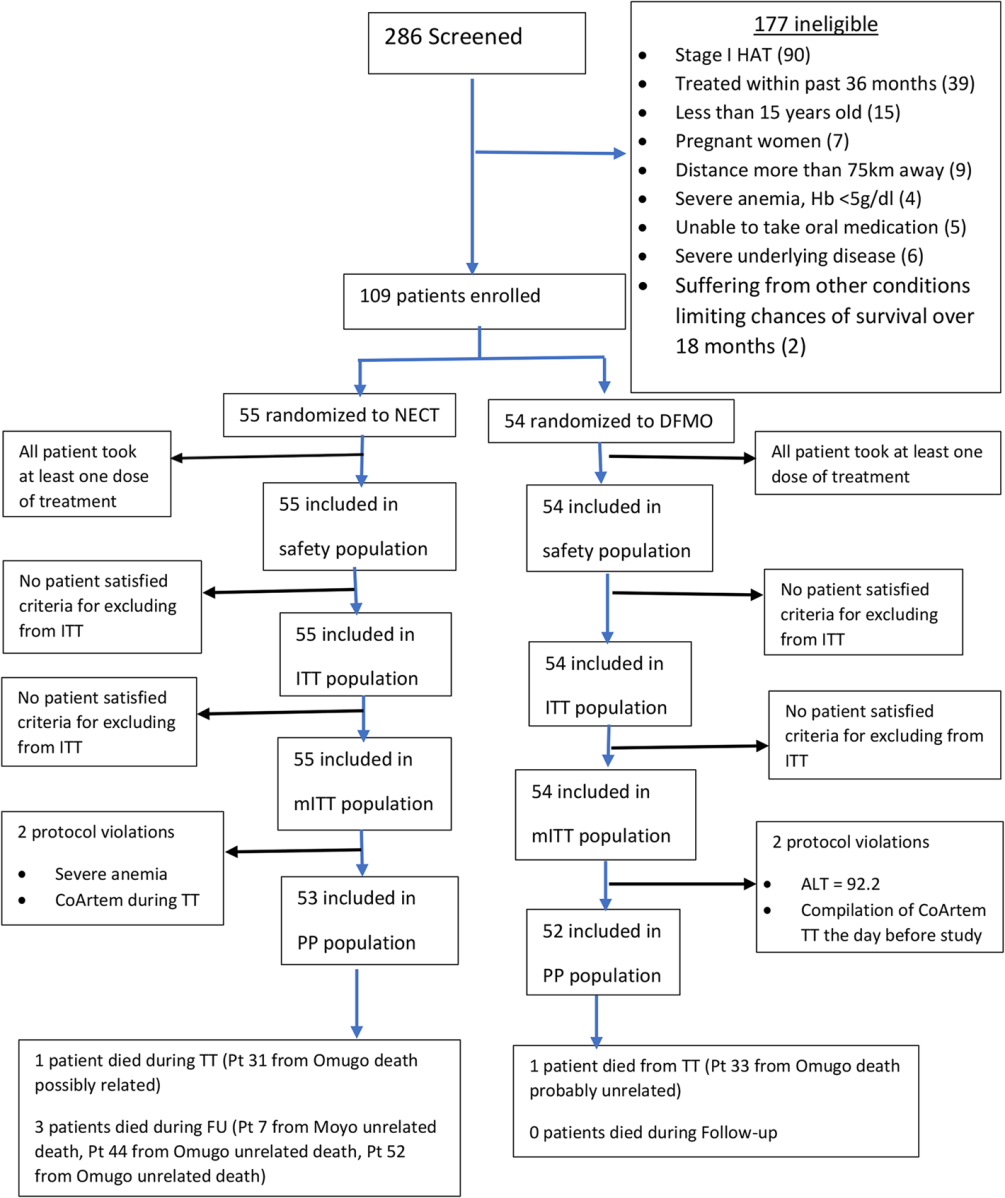
Flow diagram and treatment outcomes. *Abbreviations*: NECT, nifutimox-eflonithine combination treatment; ITT, intent-to-treat; mITT, modified ITT; PP, per-protocol

This was a randomized, controlled, open-label, non-inferiority trial. The randomisation list in blocks of ten was electronically generated at WHO/TDR and concealed from the field teams (Fig. 2 shows the trial allocation). Patients were included in chronological order as they were diagnosed. Sealed, opaque envelopes containing the treatment allocation were opened in their numerical order. Each site had an independent series.

Participants for inclusion were those 15 years of age and above, resident within 60–75 km of the health centre (depending on the site), with confirmed late stage *T. b. gambiense* HAT as demonstrated by presence of the parasites in blood, lymph node aspirates, or cerebrospinal fluid (CSF) in addition to CSF white blood cell counts > 20/μl. In addition, written informed consent had to be provided by the patient or a legally recognised guardian for minors (< 18 years) or those unable to communicate.

Excluded were pregnant women (systematic testing of urine HCG was undertaken), those treated for the same condition in the past 36 months, those unlikely to be accessible for the mandatory 18 months post-treatment follow-up, those unable to take oral medication or suffering from other conditions that would significantly limit the chances of survival through the 18 month follow-up period. Other exclusion criteria were severe anaemia (Hb < 5 g/dl), other severe underlying conditions (such as active tuberculosis, bacterial or cryptoccocal meningitis, HIV/AIDS at stages 3 or 4, as well as renal or hepatic malfunction as determined by calculated creatinine clearance < 20 ml/min, or total bilirubin > 50 μmol/l, ALAT/GPT > 70 UI/l, respectively). Patients diagnosed with *T. b. gambiense* HAT but not included in the study were treated with the standard treatment regimen on site.

### Study medications

The test treatment was nifurtimox-eflornithine combination (NECT). Nifurtimox was administered orally every 8 h, at 5 mg/kg (total daily dose = 15 mg/kg) for 10 days; eflornithine was administered for 7 days by IV infusion of 200 mg/kg every 12 h, given over 2 h (total daily dose = 400 mg/kg).

The comparator treatment was standard eflornithine treatment, given as infusions of 100 mg/kg every 6 h (total daily dose = 400 mg/kg) for 14 days.

Patients found to have malaria were treated with artemether-lumefantrine for 3 days, and the study treatment started at least 1.5 days thereafter. Treatment for other concurrent disorders was postponed to the end of hospitalisation, except if it was deemed immediately warranted. A food ration of 2100 kcal per day was provided to all study participants.

### Study procedures

All participants were hospitalised during treatment and for at least 7 days after the end of treatment and were medically assessed on a daily basis. One day after administration of the last study dose, a lumbar puncture was performed in order to determine the CSF white cell count and search for any surviving trypanosomes. Follow-up visits and laboratory evaluations were performed at 6, 12 and 18 months, to check for the presence of trypanosomes in the blood (by the haematocrit centrifugation technique) [17], CSF (by modified single centrifugation) [18], lymph node aspirates, and CSF cell count as well as IgM titres [19]. All results were confirmed by 2 independent technicians and by a third senior technician in case of discrepancies.

### Efficacy and safety evaluations

Participants were closely observed during hospitalization and scheduled for regular post-treatment follow-up for a period of 18 months. Efficacy assessment was conducted at the end of treatment and continued into the post-treatment follow-up.

Efficacy assessments performed during follow-up comprised clinical evaluation for HAT signs and symptoms, parasitological evaluations of blood, CSF and/or lymph node aspirates where applicable, as well as CSF white cell counts and IgM titres. These assessments determined the endpoint for the efficacy analyses (Table 1). A treatment failure was defined as a death during treatment due to HAT or to an adverse event related to the treatment drugs, death during follow-up that was considered likely as a consequence of HAT or to a treatment-related adverse event, death for unknown causes, non-response at EoT visit, relapse or probable relapse.

**Table 1:** Efficacy criteria

Category	Evaluation	Criteria	Time
Treatment failure	Non-responder	Trypanosomes are present in CSF at the control LP at the end of treatment	at end of treatment
Treatment failure	Relapse	Trypanosomes are present in blood, CSF or lymph at any control visit	at any control visit
Treatment failure	Probable relapse	CSF WCC has increased by > 20 cells/μl two times consecutively (regardless of IgM titre) at any control visit	at any control visit
Treatment failure	Non-responder	Death	at any control visit
Treatment failure	Relapse	Trypanosomes are present in blood, CSF or lymph	at 18 months
Treatment failure	Probable relapse	Trypanosomes are absent from blood and CSF (and lymph if adenopathy) AND CSF WCC is > 20 cells per μl	at 18 months
Cured	Not failure. Not relapse responder	Trypanosomes are absent from blood and CSF (and lymph if adenopathy) AND CSF contains ≤ 20 cells per μl	at 18 months

*Abbreviations*: *CSF* cerebrocpinal fluid, *LP* lumbar puncture, *WCC* white cell count

Safety was assessed using the National Cancer Institute Common Toxicity Criteria [20], that grades adverse events (AEs) by intensity, relation to the drug-event, and treatment outcome, serious adverse events (SAEs), and laboratory values (haematology and biochemistry).

### Analysis sets

Analysis populations were defined according to the WHO recommendations on HAT Clinical Product Development [21]. Analysis sets included: safety analysis population; intent-to-treat (ITT), per-protocol (PP) and modified ITT (mITT) efficacy analysis populations (see Table 2).

**Table 2:** Analysis sets

Analysis population	Description
Safety analysis population	All patients enrolled in the study who received at least one dose of the study medication
Intention-to-treat (ITT) population (full analysis)	All patients enrolled in the study who received at least one dose of study medication AND died during treatment or were non-responders OR reached a protocol-defined endpoint [probable relapse, relapse or death during follow-up (all causes of deaths)] OR for whom efficacy evaluation data at the test-of-cure visit (18 months) or a protocol defined earlier time-point are available
Per-protocol (PP) population	All patients enrolled in the study who: received at least one dose of study medication AND died during treatment or were non-responders OR for whom treatment was discontinued because of treatment-related adverse events OR completed the protocol-defined treatment (≥ 95%) AND reached one of the protocol-defined endpoints [probable relapse, relapse or death during follow-up (all causes of deaths except death clearly unrelated to HAT and treatment)] before the test-of-cure visit (18 months) OR have a test-of-cure visit assessment (at 18 months).
Modified full analysis (mITT) population	All patients enrolled in the study who received at least one dose of study medication AND died during treatment or were non-responders OR for whom treatment was discontinued because of treatment-related adverse events OR received a defined minimum amount of treatment (≥ 85%) AND reached a protocol-defined endpoint [probable relapse, relapse or death during follow-up (all causes of deaths)] OR for whom efficacy evaluation data at the test-of-cure visit (18 months) or a protocol defined earlier time-point are available

Lost to follow-up (LTFU) patients were defined as those for whom an evaluation at the end of treatment was available, but had no evaluation thereafter. They were included in the safety analysis.

Patients who were partially followed-up (pFU) were defined as those for whom at least one efficacy assessment after the (EoT) evaluation was available.

### Statistical analyses

A non-inferiority test was applied to the primary efficacy outcome only, i.e. the overall cure rate at 18 months after completion of treatment, as defined in Table 2, on the ITT, mITT and PP populations. Non-inferiority was confirmed if the lower limit of the 90% confidence interval of the difference in cure rates observed between the two groups was above the set non-inferiority margin (δ) of 10% (one-tailed test).

Secondary efficacy indicators were the duration of survival over the 18 month post-treatment follow-up period without laboratory signs of parasitic infection. The Kaplan-Meier analysis was performed on the ITT, mITT and PP sets. Secondary safety objectives were to compare two treatment regimens in terms of incidence of AEs by severity and relationship to treatment, proportion of patients without major AEs (grade 3 or 4, NIH/NCI Common Toxicity Criteria), incidence of adverse events leading to discontinuation, incidence of SAEs and to determine the duration of survival without laboratory signs of parasitic infection up to 18 months after treatment.

Continuous variables are here presented as mean and standard deviation and compared between treatment groups with the Student’s t-test. Otherwise, in the case where normality could not be assumed, the Mann-Whitney-Wilcoxon test was applied. Frequencies are presented as number and proportions, and compared using a Pearson’s chi-square test. If expected cell frequencies were less than 5, then the Fisher’s or Freeman-Halton (more than 2 categories) exact test was used.

Parasite-free survival up to 18 months after treatment was compared between treatment groups by using the Kaplan-Meier method and the log rank test. In the case the survival curves crossed each other, the Wilcoxon (Breslow) test was used.

All tests were two-tailed except the non-inferiority test. A *P*-value < 0.05 was considered significant. All analyses were performed with the SAS system v.9.4 (SAS Institute, Cary, NC, USA).

### Systematic review (meta-analysis)

We then carried out a metanalysis incorporating our findings to those from previous studies. We carried out a search in PubMed using the following terms: trypanosomiasis, sleeping sickness, *Trypanosoma brucei gambiense*, nifurtimox, eflornithine. We selected publications that were of randomized clinical trials or safety studies involving the use of eflornithine. For the outcome, we considered the binary endpoint as cure or failure of treatment, after follow-up of at least 6 months. Relapse cases were defined as patients with: (i) history of HAT treatment and (ii) presence of trypanosomes in lymph, blood, or CSF, or CSF leukocyte count > 20 cells/μl, having increased compared with previous count or associated with clinical features consistent with HAT. All patients who did not meet this definition at 6 or 12 months were considered cured. The number of subjects cured or failed (relapsed), together with number of subjects in each treatment group, were extracted from the selected papers for the intent to treat population.

For the statistical analysis, the risk difference was chosen to express the results. The risk difference is the difference between the observed risks with the proportion of individuals with the outcome of interest in each treatment arm. This was therefore the difference between relapse proportions of the NECT and eflornithine arms (as applies in the usual non inferiority trials). The confidence interval around the risk difference was set at 90% with a non inferiority margin of 10% (-0.1 on the forest plot X-axis). The Mantel-Haenszel method available in RevMan with a DerSimonian random effect was used.

## Results

Out of a total of 286 patients screened, 177 were excluded for reasons outlined in the patient flow cart (Fig. 2). Thus 109 participants were included in the study: 55 of these were randomized to the test group nifutimox-eflonithine (NECT) and 54 to the control group eflornithine (DMFO). Of two sites, Omugo enrolled 70% of patients (38 in the NECT and 38 in the eflornithine arm) and Moyo 30% (NECT = 17, eflornithine = 16).

Table 3 summarises the included patients’ demographic and baseline characteristics (see also Additional files 1 and 2: Tables S1 and S2 for additional baseline characteristics including laboratory and clinical findings). The baseline data were similar in the two arms, except the body mass index which was significantly higher in the eflornithine arm in the 3 efficacy analysis populations (19.8 ± 2.6 *vs* 18.4 ± 2.2 in NECT group, Student’s t-test, *t* = 2.99, *df* = 107, *P* < 0.0034, Table 3). Treatment adherence was similar in the two arms: NECT = 92.7% *vs* eflornithine = 96.3% in the ITT/mITT population, and 94.3 *vs* 96.1%, respectively in the PP population. All 109 participants were considered for the ITT, safety and mITT populations for analysis. The PP population had 105 subjects (NECT = 53, eflornithine = 52); reasons for exclusion from the PP population were major protocol violations: one case each of severe anaemia and antimalarial treatment during HAT treatment in the NECT arm, and one case each of liver enzyme abnormality (ALAT/GPT = 92.2 UI) and antimalarial treatment in the eflornithine arm. The details and breakdown are provided in Fig. 2. The 18 month cure rate was 90.9% for NECT and 88.9% for eflornithine in the ITT and mITT populations, and 90.6% for NECT and 88.5% for eflornithine in the PP population. Overall, the NECT group had no non-responders at end-of-treatment, 1 relapse and 4 deaths (1 on-treatment, 3 on follow-up), and the eflornithine group had 2 non-responders at end-of-treatment, 3 relapses and 1 death on-treatment (see below for details).

**Table 3:** Patients’ demographic and baseline characteristics by analysis set and treatment group

Demographic characteristics		ITT, mITT and safety population				PP population			
		NECT (*n* = 55)	Eflornithine (*n* = 54)	All (*n* = 109)	*P*-value	NECT (*n* = 53)	Eflornithine (*n* = 52)	All (*n* = 105)	*P*-value
Age (years)	Mean (SD)	27.22 (12.07)	27.33 (8.59)	27.28 (10.44)	0.3804^c^	27.49 (12.18)	27.38 (8.60)	27.44 (10.51)	0.4644^c^
Sex	Male, *n* (%)	29 (52.73)	28 (51.85)	57 (52.29)	0.9271^a^	28 (52.83)	26 (50.00)	54 (51.43)	0.7717^a^
	Female, *n* (%)	26 (47.27)	26 (48.15)	52 (47.71)	–	25 (47.17)	26 (50.00)	51 (48.57)	–
Height (cm)	Mean (SD)	162.6 (10.38)	165.2 (9.52)	163.9 (10.00)	0.1843^b^	162.8 (10.25)	165.1 (9.60)	164.0 (9.96)	0.2334^b^
Screening mode	Active, *n* (%)	19 (34.55)	18 (33.33)	37 (33.94)	–	19 (35.85)	17 (32.69)	36 (34.29)	0.7333^a^
	Passive, *n* (%)	36 (65.45)	36 (66.67)	72 (66.06)	–	34 (64.15)	35 (67.31)	69 (65.71)	–
Weight (kg)	Mean (SD)	49.25 (10.04)	54.18 (8.82)	51.69 (9.73)	0.0076^b^	49.58 (9.87)	54.08 (8.94)	51.80 (9.65)	0.0161^b^
BMI (kg/m^2^)	Mean (SD)	18.42 (2.23)	19.81 (2.58)	19.11 (2.50)	0.0034^b^	18.52 (2.18)	19.79 (2.63)	19.15 (2.48)	0.0086^b^
Blood pressure systolic (mmHg)	Mean (SD)	112.3 (13.97)	112.2 (14.32)	112.3 (14.08)	0.7618^c^	112.8 (14.03)	111.6 (14.21)	112.2 (14.06)	0.4247^c^
Blood pressure diastolic (mmHg)	Mean (SD)	72.22 (10.10)	73.50 (10.54)	72.85 (10.30)	0.5183^b^	72.40 (10.23)	72.92 (10.31)	72.66 (10.22)	0.8574^c^
Heart rate (/min)	Mean (SD)	82.82 (14.17)	82.19 (13.62)	82.50 (13.84)	0.8126^b^	82.57 (14.32)	82.69 (13.46)	82.63 (13.83)	0.9630^b^
Respiratory rate (/min)	Mean (SD)	20.58 (2.94)	20.69 (3.70)	20.63 (3.32)	0.7350^c^	20.49 (2.90)	20.85 (3.67)	20.67 (3.29)	0.9222^c^
Body temperature (°C)	Mean (SD)	36.53 (0.53)	36.71 (0.71)	36.62 (0.63)	0.1349^b^	36.54 (0.54)	36.73 (0.71)	36.63 (0.63)	0.1204^b^
Glasgow Coma score	Mean (SD)	14.77 (0.50)	14.83 (0.38)	14.80 (0.44)	0.8000^c^	14.77 (0.50)	14.82 (0.39)	14.79 (0.45)	0.8014^c^
Karnofsky index (%)	Mean (SD)	79.64 (9.62)	82.04 (7.62)	80.83 (8.73)	0.2976^c^	79.62 (9.80)	81.73 (7.60)	80.67 (8.80)	0.4659^c^

*Abbreviations*: *DFMO* eflornithine, *ITT* intent to treat population, *mITT* modified ITT population, *PP* per-protocol population

^a^Chi-square test

^b^Student’s t-test (pooled)

^c^Wilcoxon-Mann-Whitney test

**Fig. 3:**
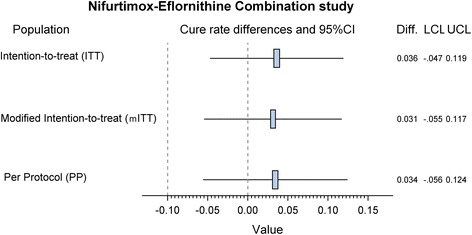
Results of the non-inferiority test for the primary efficacy by study population

Non-inferiority was demonstrated in all three analysis populations (Fig. 3) The difference in cure rates of the two groups in the ITT, mITT and PP populations was 2.02% (90% CI: -7.47–11.51%), 2.02% (90% CI: -7.47–11.51%) and 2.10% (90% CI: -7.73–11.94%), respectively. The lower limit of the 90% CI was above the non-inferiority margin.

Two patients died while on treatment (one in each arm), and three more from the NECT arm died during follow-up. Both on-treatment deaths were considered probably drug-related and counted as failure. The death on NECT occurred on day 3 of treatment following episodes of headache and vomiting; the death on eflornithine occurred on day 10 following vomiting, cellulitis in upper limb and multiple episodes of convulsions.

Two of the deaths during follow-up were due to suicide while the third was attributed to severe malaria. The first patient died 2 months after discharge. S/he had normal laboratory findings at discharge on day 17; the presumptive cause of death was reported to be malaria according to records recovered from the centre. The laboratory results of the second patient who committed suicide were within normal range except raised white blood cells (65 cells/μl) according to the results carried out on discharge - drastically reduced from 1070 cells/μl at admission. There were no facilities to carry out a *post-mortem*, neither were the reasons for suicide clearly understood.

No significant difference in time-to-relapse was found between the two study arms (Kaplan-Meier log-rank > 0.6 for the analysis sets). Significantly (*P* = 0.02) more patients (75.9%) experienced at least one laboratory adverse event in the eflornithine treatment arm than those in the NECT arm (54.6%), as shown in Table 4. Organ system drug-related adverse events did not significantly differ between the 2 arms, apart from vertigo (*P* = 0.03) and vomiting (*P* < 0.0001), which were significantly more common in the NECT arm. Laboratory assessments during hospitalisation and follow-up for the two study arms are shown in Additional file 3: Table S3 and details on all organ system drug-related adverse events, by treatment group are in the Additional file 4: Table S4.

**Table 4:** Secondary safety parameters by treatment group

	NECT (*n* = 55)	Eflornithine (*n* = 54)	All (*n* = 109)	*P*-value
	*n* (%)	*n* (%)	*n* (%)	
No. of deaths during 30 days	1 (1.8)	1 (1.9)	2 (1.8)	1.00
No. of deaths during follow-up	3 (5.5)	0	3 (2.8)	0.24
At least one severe adverse event	7 (12.7)	15 (27.8)	22 (20.2)	0.16
No. of patients with permanent treatment interruption	0	1 (1.9)	1 (0.9)	0.50
No. of patients with temporary treatment interruption	0	1 (1.9)	1 (0.9)	0.50
At least one severe adverse event (intensity grade 3 & 4) (clinical & laboratory)	14 (25.5)	15 (27.8)	29 (26.6)	0.78
At least one severe adverse event (intensity grade 3 & 4) (clinical)	9 (16.4)	13 (24.1)	22 (20.2)	0.32
At least one severe adverse event (intensity grade 3 & 4) (laboratory)	9 (16.4)	8 (14.8)	17 (15.6)	0.82
At least one adverse event (clinical & laboratory)	49 (89.1)	49 (90.7)	98 (89.9)	0.78
At least one adverse event (clinical)	44 (80.0)	46 (85.2)	90 (82.6)	0.48
At least one adverse event (laboratory)	30 (54.6)	41 (75.9)	71 (65.1)	0.02^a^
At least one adverse event (possibly) related to treatment	37 (67.3)	39 (72.2)	76 (69.7)	0.57

^a^Indicates significant differences across treatment groups

*Abbreviation*: *NECT* nifutimox-eflonithine combination treatment

For the meta-analysis, 8 studies were selected. Out of them, 4 were single arm studies with either NECT or eflornithine [14, 22–24]. One clinical trial compared NECT with melarsoprol [10] and was used only in a sensitivity analysis. The primary analysis contained 3 clinical trials of NECT *vs* eflornithine [11, 25, this study]; 250 patients in the NECT arm to 248 patients in the eflornithin arm have been included in the analysis.

**Fig. 4:**
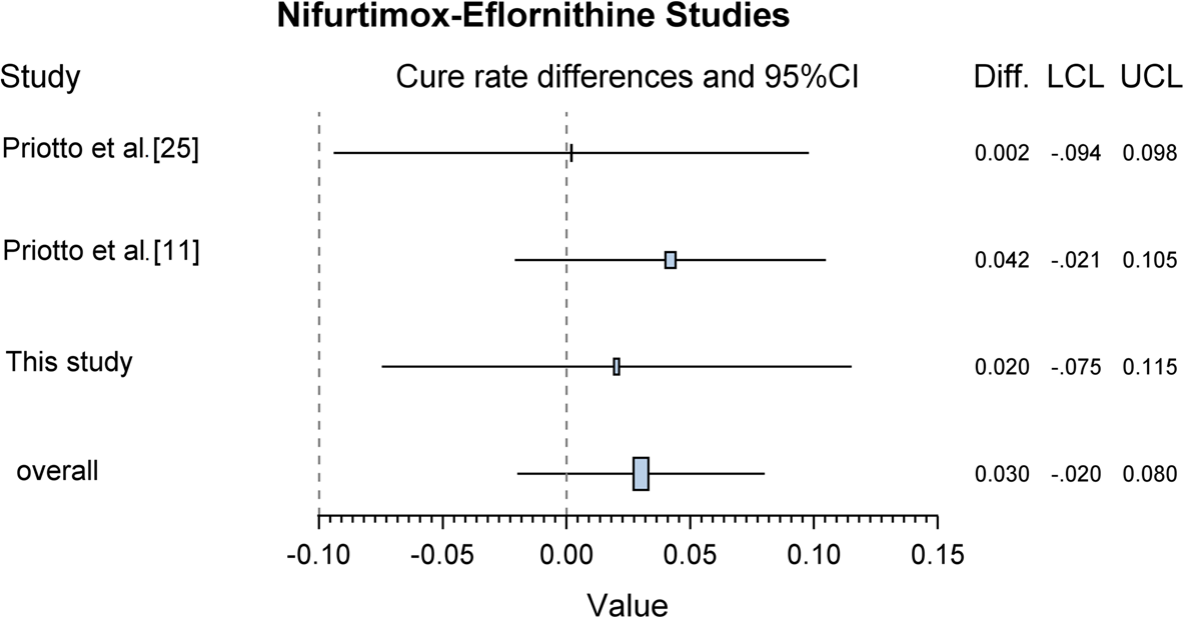
Forest plot of randomised controlled trials of second-stage HAT treatment comparing nifutimox-eflonithine *vs* eflornithine

In the primary analysis, a difference of 3% was found (Fig. 4). The lower limit of the 90% confidence interval was -2% thus higher than the non-inferiority margin of 10% (RD = 3%; 90% CI: -2–7%).

**Fig. 5:**
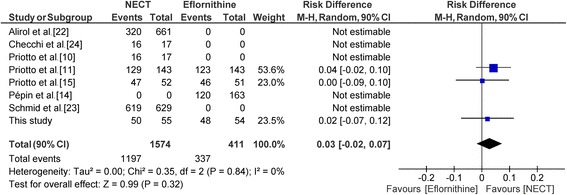
Forest plot of sensitivity analysis of 3 clinical trials comparing NECT to eflornithine and 1 clinical trial comparing NECT to melarsoprol. (Risk difference, random effects model)

A sensitivity analysis (Fig. 5) was performed by adding the results of the Priotto et al. [10] study with melarsoprol + eflornithine as the comparator (this study did not include eflornithine monotherapy as a comparator). The meta analytic estimates of the risk difference was RD = 4% (90% CI: -1–8%).

## Discussion

This study shows that a 10-day course with a combination treatment of oral nifurtimox and parenteral eflornithine (NECT) is non-inferior to the standard 2-week course with parenteral eflornithine, and was well tolerated. The cure rate 18 months after starting treatment was about 91% in the NECT and 89% in the eflornithine group in all analysis sets.

Recruitment in this study was lower than anticipated for a number of factors, but mostly due to a drastic reduction in *T. b. gambiense* HAT incidence following mass population screening in years preceding the study. In this study, active surveillance was undertaken in order to boost enrolment, but 177 potential participants were found to be ineligible. More than half of these (99) were first-stage HAT patients, suggesting that after the previous screening activities many of the cases detected were recent infections. Notably, while we could not reach the planned sample size, the data generated from this trial are of value as they complement prior findings and increase the available dataset of closely monitored second-stage HAT treatments.

**Fig. 6:**
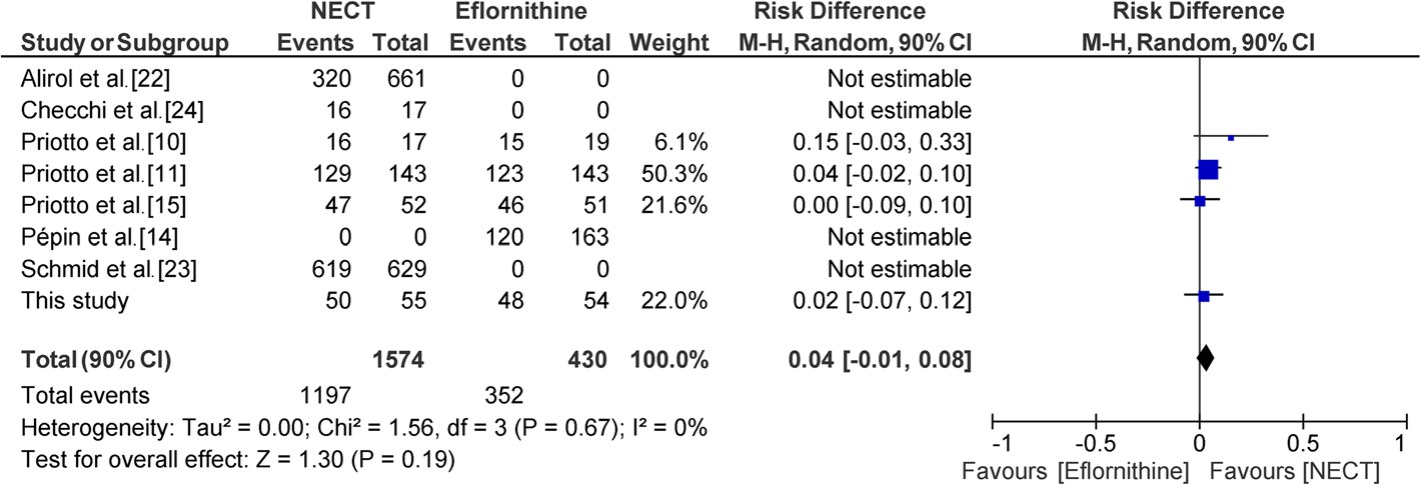
Forest plot from meta-analysis of 3 clinical trials comparing NECT to eflornithine. (Risk difference, random effects model)

It is important to consider the outcome of this trials in the context of other studies of NECT. The cure rates obtained in the present trial are comparable to those reported in previous randomised controlled trials and patient series [10, 11, 15, 24] as shown in Table 5. In addition, when combining the results of the present trial with the two other available randomised controlled trials [11, 15], the risk differences between the cure rates of the NECT and eflornithine treatment are remarkably similar. The calculated overall risk difference of 0.03 (95% CI: -0.02–0.07) is clearly in favour of NECT (see Fig. 6).

**Table 5:** Comparison of NECT cure rates in clinical trials and case series conducted to date

Reference	Test of cure	NECT			Eflornithine			Difference	
		*n*	%	95% CI	*n*	%	95% CI	%	95% CI
Priotto et al. [15]	Treated	52	–	–	51	–	–	–	–
	Cure	47	90.4	80.5–100.3	46	90.2	80.1–100.3	0.2	-9.4–9.8
Priotto et al. [12]	Treated	143	–	–	143	–	–	–	–
	Cure	129	90.2	84.7–95.8	123	86.0	79.6–92.4	4.2	-2.1–10.5
	Cure or probable cure	138	96.5	92.8–100.2	131	91.6	86.4–96.8	4.9	0.3–9.5
This study	Treated	55	–	–	54	–	–	–	–
	Cure	50	90.9	81.5–100.3	48	88.9	78.7–99.1	2.0	-7.5–11.5
Priotto et al. [11]	Treated	31	–	–	–	–	–	–	–
	Cure	29	93.5	81.7–105.4	–	–	–	–	–
Checchi et al. [24]	Treated	17				–	–	–	–
	Cure	16	94.1	77.2–111.1		–	–	–	–

*Abbreviation*: *CI* confidence interval

A previous systematic review [25] of randomised clinical trials of HAT treatments was not particularly helpful in the present context, as outcomes from only one NECT *vs* eflornithine trial [11] were considered. In the present meta-analysis, we have included as many studies as possible (not so many are available) although we could not utilize the entire sets for all analyses due to inherent limitations. Nevertheless, NECT was found to exhibit non-inferiority to eflornithine to diminish the number of failures in the treatment of *T. b. gambiense* HAT. Indeed, both the primary and the sensitivity analyses resulted in an estimate of the lower limit of the 90% CI above the non-inferiority margin.

While the standard eflornithine regimen requires 6-hourly infusions given its short half-life, this study has demonstrated eflornithine in 12-hourly infusions is highly effective when administered in combination with oral nifurtimox. Given the short half-life of eflornithine, 6-hourly infusions are required to sustain its trypanostatic effect. Our results confirm that 12-hourly eflornithine infusions are highly effective alongside oral nifurtimox.

There are potential limitations to this study. One relates to the use of an open-label design which was unavoidable as a blinded design is unacceptable given the different modes of administration of nifurtimox (oral) and eflornithine (6-hourly for 14 days or 12-hourly for 7 days infusion) would require a double dummy design and thus 42 placebo infusions of 2 h duration in the patients on NECT. The impact of this limitation on the study outcome is, however, minimal as the efficacy outcomes were majorly inferred from laboratory analyses confirmed by two independent readers. The other potential limitation relates to the safety analysis where the duration of hospitalization was different for the two groups due to the different treatment schedules. A longer hospitalization time means a longer period of observation and recording of more adverse events.

The major challenges faced in this trial include failure to recruit the desired number of patients in the calculated sample size, logistical hindrances associated with delivery of internationally procured supplies, high mobility of patients with some moving across international borders thus complicating follow up, and limited infrastructure/research capacity in rural Uganda where the study was located. Despite these challenges the trial achieved over 90% end of follow up assessment at 18 months and the established trial methods remained in firm compliance with GCP guidelines.

In summary, this study confirms earlier findings supporting the recommendations of NECT for first-line treatment of late stage *T. b. gambiense* infections. NECT continues to be used in several centres and is generally believed to have improved prognosis of treated patients; the only issue being the above average nursing requirement [26].

## Conclusions

Our study has confirmed findings from earlier clinical trials and support implementation of NECT as first-line treatment for late stage *T. b. gambiese* HAT. The NECT regimen is simpler, safer, shorter and less expensive than single-agent DFMO. It therefore provides a relaible alternative for treatment as the search for new chemotherapeutic agents continues.

## Additional files

Additional file 1: **Table S1.** Baseline characteristics of modified intention-to-treat population categorized by treatment. (DOCX 18 kb)

Additional file 2: **Table S2.** Baseline characteristics of per-protocol (PP) population categorized by treatment. (DOCX 20 kb)

Additional file 3: **Table S3.** Laboratory assessments per treatment group. (DOCX 22 kb)

Additional file 4: **Table S4.** Organ system drug-related adverse events, by treatment group. (DOCX 21 kb)

## Abbreviations

AE: Adverse event
AIDS: Acquired immune deficiency syndrome
ALAT/GPT: Alanine amino transferase/ glutamic pyruvic transaminase
BTT: Bi-therapy trial
CI: Confidence interval
CSF: Cerebrospinal fluid
DFMO: Difluoromethylornithine
EoT: End of treatment
GCP/GLP: Good clinical practices/ good laboratory practices
HAT: Human african trypanosomiasis
HCG: Human chorionic gonadotropin
HIV: Human immune-deficiency virus
IgM: Immuneglobulin
ITT: Intent to treat
LFTU: Lost to follow-up
LP: Lumbar puncture
mITT: Modified intent to treat
NECT: Nifurtimox-eflornithine combination treatment
NGO: Non-governmental organizations
NIH/NCI: National Institutes of Health / National Cancer Institute
pFU: Partially followed-up
PP: Per-protocol
SAE: Serious adverse event
TDR: Special Programme for Research and Training in Tropical Diseases
WHO: World Health Organization
