# Supplementary material for: A multicentre, randomised, non-inferiority clinical trial comparing a nifurtimox-eflornithine combination to standard eflornithine monotherapy for late stage Trypanosoma brucei gambiense human African trypanosomiasis in Uganda
Source: Parasit Vectors. 2018 Feb 22;11:105. doi: 10.1186/s13071-018-2634-x (PMC5824494; doi:10.1186/s13071-018-2634-x)
Supplement: Supplementary file 1 — Table S1. Baseline characteristics of modified intention-to-treat population categorized by treatment. (DOCX 18 kb) [file 13071_2018_2634_MOESM1_ESM.docx]

**Additional file 1: Table S1**. Baseline characteristics of modified Intention-to-treat Population categorized by treatment.

|  | NECT (N=55) | DFMO (N=54) | All (N=109) | P-value |
| --- | --- | --- | --- | --- |
| **Parasitological findings** |  |  |  |  |
| Presence of trypanosomes |  |  |  |  |
| In lymph nodes | 28 (50.9%) | 29 (53.7%) | 57 (52.3%) |  |
| In blood | 39 (70.9%) | 39 (72.2%) | 78 (71.2%) |  |
| In CSF | 43 (78.2%) | 40 (70.1%) | 83 (76.2%) |  |
| CSF WBC count (median, cells per µl) | 212 | 202 | 203 |  |
| Categorization of WBC |  |  |  |  |
| 6-20 | 1 (1.8%) | 0 | 1 (0.9) |  |
| 21-100 | 14 (25.5%) | 17 (31.5%) | 31 (28.4%) |  |
| >100 | 40 (72.7%) | 37 (68.5%) | 77 (70.6%) |  |
| CSF IgM titre (median) | 64 | 64 | 64 |  |
|  |  |  |  |  |
| **Clinical characteristics** |  |  |  |  |
| Headache | 45(81.82%) | 47(87.04%) | 92(84.40%) | 0.45 |
| Pruritus | 37(67.27%) | 31(57.41%) | 68(62.39%) | 0.29 |
| Tremor | 16(29.09%) | 12(22.22%) | 28(25.69%) | 0.41 |
| Speech impairment | 6(10.91%) | 3(5.556%) | 9(8.257%) | 0.49 |
| Abnormal Movements | 12(21.82%) | 6(11.11%) | 18(16.51%) | 0.13 |
| Lymphadenopathy | 29(52.73%) | 31(57.41%) | 60(55.05%) | 0.62 |
| Insomnia: Day-time Sleep | 27(49.09%) | 21(38.89%) | 48(44.04%) | 0.28 |
| Insomnia: Night-time Sleep | 9(16.36%) | 6(11.11%) | 15(13.76%) | 0.43 |
| Walking Disability | 6(10.91%) | 3(5.556%) | 9(8.257%) | 0.49 |
| General Motor Weakness | 7(12.73%) | 13(24.07%) | 20(18.35%) | 0.13 |
| Unusual behavior | 15(27.27%) | 13(24.07%) | 28(25.69%) | 0.70 |
| Inactivity | 9(16.36%) | 9(16.67%) | 18(16.51%) | 0.97 |
| Aggressivity | 4(7.273%) | 1(1.852%) | 5(4.587%) | 0.36 |
| Disturbance of Menstrual Cycle  (FemaleS only) | 7(12.73%) | 9(16.67%) | 16(14.68%) | 0.56 |
| Anaemia | 35(63.64%) | 34(62.96%) | 69(63.30%) | 0.94 |
| Diarrhoea | 1(1.818%) | 0 | 1(0.917%) | 1.00 |
| Malaise | 17(30.91%) | 17(31.48%) | 34(31.19%) | 0.95 |
| Blood pressure systolic (mmHg) | 112.3±13.97 | 112.2±14.32 | 112.3±14.08 | 0.76 |
| Blood pressure Diastolic (mmHg) | 72.22±10.10 | 73.5±10.54 | 72.85±10.30 | 0.52 |
| Heart rate (/min) | 82.82±14.17 | 82.19±13.62 | 82.5±13.84 | 0.81 |
| Respiratory rate (/min) | 20.58±2.94 | 20.69±3.7 | 20.63±3.32 | 0.74 |
| Body Temperature (^o^C) | 36.53±0.53 | 36.71±0.71 | 36.62±0.63 | 0.13 |
| Glasgow Coma Score | 14.77±0.5 | 14.83±0.38 | 14.8±0.44 | 0.8 |
| Karnofsky Index (%) | 79.64±9.62 | 82.04±7.62 | 80.83±8.73 | 0.3 |
| Others 1 | 29(52.73%) | 32(59.26%) | 61(55.96%) | 0.71 |
| Others 2 | 4(7.273%) | 15(27.78%) | 19(17.43%) | 1.00 |

NECT = nifutimox-eflonithine combination treatment. DMFO = difluoromethylornithine. * indicates significant differences across treatment groups. Means are presented as mean±SD.
