# Supplementary material for: A multicentre, randomised, non-inferiority clinical trial comparing a nifurtimox-eflornithine combination to standard eflornithine monotherapy for late stage Trypanosoma brucei gambiense human African trypanosomiasis in Uganda
Source: Parasit Vectors. 2018 Feb 22;11:105. doi: 10.1186/s13071-018-2634-x (PMC5824494; doi:10.1186/s13071-018-2634-x)
Supplement: Supplementary file 2 — Table S2. Baseline characteristics of per-protocol (PP) population categorized by treatment. (DOCX 20 kb) [file 13071_2018_2634_MOESM2_ESM.docx]

**Additional file 2: Table S2**. Baseline characteristics of Per Protocol (PP) Population categorized by treatment.

|  | NECT (N=53) | DFMO (N=52) | All (N=105) | P-value |
| --- | --- | --- | --- | --- |
| **Demographic characteristics** |  |  |  |  |
| Age | 27.49±12.18 | 27.38±8.6 | 27.44±10.51 | 0.46 |
| Sex |  |  |  |  |
| Male | 28 (52.8%) | 26 (50%) | 54 (51.4%) | 0.77 |
| Female | 25 (47.2%) | 26 (50%) | 51 (48.5%) |  |
| Height | 162.8±10.25 | 165.1±9.5 | 164±1.0 | 0.23 |
| Screening mode |  |  |  |  |
| Active | 19 (35.9%) | 17 (32.7%) | 36 (34.3%) | 0.73 |
| Passive | 34 (64.2%) | 35 (67.3%) | 69 (65.7%) |  |
| Weight (kg) | 49.58±9.87 | 54.08±8.94 | 51.80±9.65 | 0.02 |
| BMI (kg/m2) | 18.52±2.18 | 19.79±2.63 | 19.15±2.48 | 0.00* |
|  |  |  |  |  |
| **Parasitological findings** |  |  |  |  |
| Presence of trypanosomes |  |  |  |  |
| In lymph nodes | 26 (49.1%) | 28 (53.9%) | 54 (51.4%) |  |
| In blood | 37 (69.8%) | 38 (73.1%) | 75 (71.4%) |  |
| In CSF | 41 (77.4%) | 39 (75%) | 80 (76.2%) |  |
| CSF WBC count (median, cells per µl) | 200 | 203 | 203 |  |
| Categorization of WBC |  |  |  |  |
| 6-20 | 1 (1.9%) | 0 | 1 (1.0%) |  |
| 21-100 | 14 (26.4%) | 16 (30.8%) | 30 (28.6%) |  |
| >100 | 38 (71.7%) | 36 (69.2%) | 74 (70.5%) |  |
| CSF IgM titre (median) | 96 | 64 | 64 |  |
|  |  |  |  |  |
| **Clinical characteristics** |  |  |  |  |
| Headache | 43(81.13%) | 45(86.54%) | 88(83.81%) | 0.45 |
| Pruritus | 36(67.92%) | 31(59.62%) | 67(63.81%) | 0.38 |
| Tremor | 15(28.30%) | 11(21.15%) | 26(24.76%) | 0.40 |
| Speech impairment | 6(11.32%) | 3(5.769%) | 9(8.571%) | 0.49 |
| Abnormal Movements | 12(22.64%) | 6(11.54%) | 18(17.14%) | 0.13 |
| Lymphadenopathy | 28(52.83%) | 30(57.69%) | 58(55.24%) | 0.62 |
| Insomnia: Day-time Sleep | 26(49.06%) | 20(38.46%) | 46(43.81%) | 0.27 |
| Insomnia: Night-time Sleep | 8(15.09%) | 5(9.615%) | 13(12.38%) | 0.39 |
| Walking Disability | 6(11.32%) | 3(5.769%) | 9(8.571%) | 0.49 |
| General Motor Weakness | 7(13.21%) | 12(23.08%) | 19(18.10%) | 0.19 |
| Unusual behavior | 15(28.30%) | 13(25.00%) | 28(26.67%) | 0.70 |
| Inactivity | 9(16.98%) | 9(17.31%) | 18(17.14%) | 0.96 |
| Aggressivity | 4(7.547%) | 1(1.923%) | 5(4.762%) | 0.36 |
| Disturbance of Menstrual Cycle  (FemaleS only) | 7(13.21%) | 9(17.31%) | 16(15.24%) | 0.56 |
| Anaemia | 33(62.26%) | 33(63.46%) | 66(62.86%) | 0.90 |
| Diarrhoea | 1(1.887%) |  | 1(0.952%) | 1.00 |
| Malaise | 16(30.19%) | 17(32.69%) | 33(31.43%) | 0.78 |
| Blood pressure systolic (mmHg) | 112.8±14.03 | 111.6±14.21 | 112.2±14.06 | 0.42 |
| Blood pressure Diastolic (mmHg) | 72.4±10.23 | 72.92±10.31 | 72.66±10.22 | 0.86 |
| Heart rate (/min) | 82.57±14.32 | 82.69±13.46 | 82.63±13.83 | 0.96 |
| Respiratory rate (/min) | 20.49±2.9 | 20.85±3.67 | 20.67±3.29 | 0.92 |
| Body Temperature (^o^C) | 36.54±0.54 | 36.73±0.71 | 36.63±0.63 | 0.12 |
| Glasgow Coma Score | 14.77±0.5 | 14.82±0.39 | 14.79±0.45 | 0.8 |
| Karnofsky Index (%) | 79.62±9.8 | 81.73±7.6 | 80.67±8.8 | 0.47 |
| Others 1 | 27(50.94%) | 32(61.54%) | 59(56.19%) | 0.49 |
| Others 2 | 4(7.547%) | 15(28.85%) | 19(18.10%) | 1.00 |
|  |  |  |  |  |

NECT = nifutimox-eflonithine combination treatment. DMFO = difluoromethylornithine. * indicates significant differences across treatment groups. Means are presented as mean±SD.
