# Supplementary material for: A multicentre, randomised, non-inferiority clinical trial comparing a nifurtimox-eflornithine combination to standard eflornithine monotherapy for late stage Trypanosoma brucei gambiense human African trypanosomiasis in Uganda
Source: Parasit Vectors. 2018 Feb 22;11:105. doi: 10.1186/s13071-018-2634-x (PMC5824494; doi:10.1186/s13071-018-2634-x)
Supplement: Supplementary file 3 — Table S3. Laboratory assessments per treatment group. (DOCX 22 kb) [file 13071_2018_2634_MOESM3_ESM.docx]

**Additional file 3: Table S3.** Laboratory assessments per treatment group.

|  | NECT (N=55) | DFMO (N=54) | All (N=109) |
| --- | --- | --- | --- |
| Haematology |  |  |  |
| Haemoglobin |  |  |  |
| D11/15 | 10.95±2.20) | 10.48±1.58 | 10.72±1.93 |
| D17/21 | 11.42±1.77 | 10.78±1.31 | 11.10±1.59 |
| Follow-up on M6 | 12.44±1.64 | 13.07±1.66 | 12.76±1.67 |
| Follow-up on M12 | 12.35±1.62 | 12.25±1.69 | 12.30±1.63 |
| Follow-up on M18 | 12.42±1.53 | 11.70±1.40 | 12.11±1.49 |
| Change Baseline-D11/15 | 16.43±1.17) | 8.00±1.2 | 4.45± (8.4) |
| Change Baseline-D17/21 | 21.48±1.27 | 5.86±1.3 | 8.09± 9.1.6 |
| White blood cell (WBC) |  |  |  |
| D11/15 | 6982±2953 | 6224±2633 | 6610±2813 |
| D17/21 | 7287±3306 | 7923±3498 | 7599±3399 |
| Follow-up on M6 | 5796±3622 | 6538±1391 | 6252±2376 |
| Follow-up on M12 | 6000±2111 | 6300±282.8 | 6100±1647 |
| Follow-up on M18 | ND | ND | ND |
| Change Baseline-D11/15 | 15.40±45.76 | 7.26±41.79 | 11.41± 43.84 |
| Change Baseline-D17/21 | 19.31± 53.22 | 38.58± 59.19 | 28.75± 56.76 |
| Neutrophil |  |  |  |
| D11/15 | 2519±1321 | 2432±1754 | 2476±1541 |
| D17/21 | 2536±1442 | 3059±1791 | 2793±1635 |
| Follow-up on M6 | 1465±1026 | 2332±662.9 | 2017±878.1 |
| Follow-up on M12 | 1470±1331 | 2272±280.0 | 1791±1048 |
| Follow-up on M18 | ND | ND | ND |
| Change Baseline-D11/15 | 32.18± 90.72 | 9.66±84.59 | 21.13± 88.08 |
| Change Baseline-D17/21 | 37.45±121.9 | 33.49±82.19 | 35.51±103.8 |
| lymphocytes |  |  |  |
| D11/15 | 3643±1554 | 3247±1683 | 3449±1623 |
| D17/21 | 3835±1907) | 4058±1942) | 3944±1917 |
| Follow-up on M6 | 2844±2123 | 3124±971.8 | 3023±1392 |
| Follow-up on M12 | 1976±1741 | 3579±1006 | 2617±1594 |
| Follow-up on M18 | ND | ND | ND |
| Change Baseline-D11/15 | ND | ND | ND |
| Change Baseline-D17/21 | ND | ND | ND |
| Monocytes |  |  |  |
| D11/15 | 288.2±328.8 | 243.9±179.5 | 266.4±265.9 |
| D17/21 | 332.0±291.5 | 369.0±348.7 | 350.1±319.7 |
| Follow-up on M6 | 361.4± 357.7 | 468.5± 250.0 | 429.6±280.7 |
| Follow-up on M12 | 47.17±43.78) | 215.5±212.8 | 114.5±144.2 |
| Follow-up on M18 | ND | ND | ND |
| Change Baseline-D11/15 | ND | ND | ND |
| Change Baseline-D17/21 | ND | ND | ND |
| Basophils |  |  |  |
| D11/15 | 4.57±17.78 | 4.90±14.79 | 4.73±16.31 |
| D17/21 | 14.73±35.89 | 13.36±44.78 | 14.06±40.28 |
| Follow-up on M6 | 0.00 | 7.93±20.98 | 5.05±16.73 |
| Follow-up on M12 | 18.33±31.75 | 30.50±43.13 | 23.20±31.84 |
| Follow-up on M18 | ND | ND | ND |
| Change Baseline-D11/15 |  |  |  |
| Change Baseline-D17/21 |  |  |  |
| Biochemistry |  |  |  |
| Total bilirubin |  |  |  |
| D11/15 | 7.12±4.28 | 9.35±10.54 | 8.21±8.03 |
| D17/21 | 9.88±4.86 | 9.99±3.39 | 9.94±4.08 |
| Follow-up on M6 | 15.39±0.00 | 19.67±1.21 | 17.53±2.56 |
| Follow-up on M12 | 22.80±14.54 | 18.81±7.25 | 21.20±11.12 |
| Follow-up on M18 | ND | ND | ND |
| Change Baseline-D11/15 | ND | ND | ND |
| Change Baseline-D17/21 | ND | ND | ND |
| Alanine transferase |  |  |  |
| D11/15 | 6.40±5.85 | 7.86±9.21 | 7.12±7.68 |
| D17/21 | 16.62±15.43 | 19.75±24.53 | 18.24±20.33 |
| Follow-up on M6 | 35.15±9.40 | 10.55±4.45 | 22.85±15.42 |
| Follow-up on M12 | 19.23±14.59 | 23.40±9.19 | 20.90±11.52 |
| Follow-up on M18 | ND | ND | ND |
| Change Baseline-D11/15 | ND | ND | ND |
| Change Baseline-D17/21 | ND | ND | ND |
| Creatinine |  |  |  |
| D11/15 | 45.77±17.99 | 45.24±18.49 | 45.51±18.15 |
| D17/21 | 57.46±21.27 | 54.81±24.33 | 56.08±22.51 |
| Follow-up on M6 | ND | 48.62±6.25 | 48.62±6.25 |
| Follow-up on M12 | 61.88±8.84 | 97.24±0.00 | 70.72±19.10 |
| Follow-up on M18 | ND | ND | ND |
| Change Baseline-D11/15 | ND | ND | ND |
| Change Baseline-D17/21 | ND | ND | ND |
| Creatinine clearance |  |  |  |
| D11/15 | 198.4±84.43 | 225.2±104.7 | 211.6±95.42 |
| D17/21 | 150.5±65.71 | 214.5±109.8 | 183.7±95.33 |
| Follow-up on M6 | 0 | 160.9±33.01 | 160.9±33.01 |
| Follow-up on M12 | 134.3±55.05 | 86.66±0 .00 | 122.4±50.88 |
| Follow-up on M18 | ND | ND | ND |
| Change Baseline-D11/15 | ND | ND | ND |
| Change Baseline-D17/21 | ND | ND | ND |
|  |  |  |  |

NECT = nifutimox-eflonithine combination treatment. DMFO = difluoromethylornithine. D and M mean days and months respectively. * indicates significant differences across treatment groups. Means are presented as mean±SD
