# Supplementary material for: A multicentre, randomised, non-inferiority clinical trial comparing a nifurtimox-eflornithine combination to standard eflornithine monotherapy for late stage Trypanosoma brucei gambiense human African trypanosomiasis in Uganda
Source: Parasit Vectors. 2018 Feb 22;11:105. doi: 10.1186/s13071-018-2634-x (PMC5824494; doi:10.1186/s13071-018-2634-x)
Supplement: Supplementary file 4 — Table S4. Organ system drug-related adverse events, by treatment group. (DOCX 21 kb) [file 13071_2018_2634_MOESM4_ESM.docx]

**Additional file 4: Table** **S4.** Organ system drug-related adverse events, by treatment group

|  | NECT  (N=55) | eflornithine (N=54) | All  (N=109) | P-value |
| --- | --- | --- | --- | --- |
| **Blood & lymphatic disorders** |  |  |  |  |
| Anaemia | 2 (3.64%) | 6 (11.1%) | 8 (7.34%) | 0.16 |
| Neutropenia | 1 (1.82%) | 2 (3.70%) | 3 (2.75%) | 0.56 |
| **Cardiac disorders** |  |  |  |  |
| Bradycardia | 3 (5.45%) | 4 (7.41%) | 7 (6.42%) | 0.71 |
| Tachycardia | 3 (5.45%) | 1 (1.85%) | 4 (3.67%) | 0.32 |
| **Ear and labyrinth disorders** |  |  |  |  |
| Vertigo | 4 (7.27%) | 0 | 4 (3.67%) | 0.03* |
| **Eye disorders** |  |  |  |  |
| Conjunctivitis | 1 (1.82%) | 1 (1.85%) | 2 (1.83%) | 1.00 |
| **Gastrointestinal disorders** |  |  |  |  |
| Abdominal discomfort | 2 (3.64%) | 1 (1.85%) | 3 (2.75%) | 0.56 |
| **Abdominal pain** | 6 (10.9%) | 10 (18.5%) | 16 (14.7%) | 0.32 |
| **Abdominal pain upper** | 0 | 4 (7.41%) | 4 (3.67%) | 0.03* |
| **Constipation** | 0 | 1 (1.85%) | 1 (0.92%) | 0.21 |
| Diarrhoea | 6 (10.9%) | 7 (13.0%) | 13 (11.9%) | 0.78 |
| Dyspepsia | 4 (7.27%) | 1 (1.85%) | 5 (4.59%) | 0.18 |
| Inguinal hernia | 1 (1.82%) | 0 | 1 (0.92%) | 0.21 |
| Nausea | 14 (25.5%) | 6 (11.1%) | 20 (18.3%) | 0.07 |
| Stomatitis | 0 | 1 (1.85%) | 1 (0.92%) | 0.21 |
| Vomiting | 21 (38.2%) | 4 (7.41%) | 25 (22.9%) | 0.00* |
| **General disorders** |  |  |  |  |
| Chest pain | 1 (1.82%) | 3 (5.56%) | 4 (3.67%) | 0.32 |
| Chills | 0 | 1 (1.85%) | 1 (0.92%) | 0.21 |
| Injection site pain | 1 (1.82%) | 1 (1.85%) | 2 (1.83%) | 1.00 |
| Injection site reaction | 1 (1.82%) | 5 (9.26%) | 6 (5.50%) | 0.10 |
| Oedema | 0 | 1 (1.85%) | 1 (0.92%) | 0.21 |
| Oedema peripheral | 1 (1.82%) | 0 | 1 (0.92%) | 0.21 |
| Pain | 1 (1.82%) | 1 (1.85%) | 2 (1.83%) | 1.00 |
| Puncture site pain | 1 (1.82%) | 0 | 1 (0.92%) | 0.21 |
| Pyrexia | 9 (16.4%) | 17 (31.5%) | 26 (23.9%) | 0.12 |
| Injection site swelling | 0 | 1 (1.85%) | 1 (0.92%) | 0.21 |
| **Infections and infestations** |  |  |  |  |
| Abscess | 0 | 1 (1.85%) | 1 (0.92%) | 0.21 |
| Bronchopneumonia | 0 | 1 (1.85%) | 1 (0.92%) | 0.21 |
| Cellulitis | 1 (1.82%) | 3 (5.56%) | 4 (3.67%) | 0.32 |
| Device related infection | 0 | 2 (3.70%) | 2 (1.83%) | 0.10 |
| Gastroenteritis | 0 | 1 (1.85%) | 1 (0.92%) | 0.21 |
| Giardiasis | 2 (3.64%) | 0 | 2 (1.83%) | 0.10 |
| Helminthic infection | 1 (1.82%) | 0 | 1 (0.92%) | 0.21 |
| Infection | 1 (1.82%) | 0 | 1 (0.92%) | 0.21 |
| Malaria | 6 (10.9%) | 9 (16.7%) | 15 (13.8%) | 0.44 |
| Otitis media chronic | 0 | 2 (3.70%) | 2 (1.83%) | 0.10 |
| Pharyngitis | 1 (1.82%) | 0 | 1 (0.92%) | 0.211 |
| Typhoid fever | 1 (1.82%) | 1 (1.85%) | 2 (1.83%) | 1.00 |
| Upper respiratory tract infection | 1 (1.82%) | 1 (1.85%) | 2 (1.83%) | 1.00 |
| Urinary tract infection | 0 | 1 (1.85%) | 1 (0.92%) | 0.21 |
| Wound infection | 0 | 1 (1.85%) | 1 (0.92%) | 0.21 |
| Wound sepsis | 1 (1.82%) | 1 (1.85%) | 2 (1.83%) | 1.00 |
| **Injury, poisoning and procedural complications** |  |  |  |  |
| Soft tissue injury | 0 | 1 (1.85%) | 1 (0.92%) | 0.21 |
| **Metabolism and nutrition disorders** |  |  |  |  |
| Decreased appetite | 12 (21.8%) | 5 (9.26%) | 17 (15.6%) | 0.09 |
| **Musculoskeletal and connective tissue disorders** |  |  |  |  |
| Arthralgia | 0 | 2 (3.70%) | 2 (1.83%) | 0.10 |
| Arthritis | 0 | 2 (3.70%) | 2 (1.83%) | 0.10 |
| Back pain | 3 (5.45%) | 3 (5.56%) | 6 (5.50%) | 1.00 |
| Myositis | 0 | 1 (1.85%) | 1 (0.92%) | 0.21 |
| Neck pain | 0 | 2 (3.70%) | 2 (1.83%) | 0.10 |
| Pain in extremity | 1 (1.82%) | 1 (1.85%) | 2 (1.83%) | 1.00 |
| **Nervous system disorders** |  |  |  |  |
| Ataxia | 1 (1.82%) | 0 | 1 (0.92%) | 0.21 |
| Convulsion | 7 (12.7%) | 3 (5.56%) | 10 (9.17%) | 0.21 |
| Dizziness | 2 (3.64%) | 1 (1.85%) | 3 (2.75%) | 0.56 |
| Headache | 8 (14.5%) | 8 (14.8%) | 16 (14.7%) | 1.00 |
| Tremor | 0 | 1 (1.85%) | 1 (0.92%) | 0.21 |
| **Psychiatric disorders** |  |  |  |  |
| Abnormal behaviour | 1 (1.82%) | 0 | 1 (0.92%) | 0.21 |
| Confusional state | 1 (1.82%) | 2 (3.70%) | 3 (2.75%) | 0.56 |
| Hallucination | 5 (9.09%) | 1 (1.85%) | 6 (5.50%) | 0.10 |
| hallucination, visual | 1 (1.82%) | 1 (1.85%) | 2 (1.83%) | 1.00 |
| **Respiratory, thoracic and mediastinal disorders** |  |  |  |  |
| Asthma | 1 (1.82%) | 0 | 1 (0.92%) | 0.21 |
| Cough | 2 (3.64%) | 4 (7.41%) | 6 (5.50%) | 0.41 |
| Epistaxis | 2 (3.64%) | 1 (1.85%) | 3 (2.75%) | 0.56 |
| **Skin and subcutaneous tissue disorders** |  |  |  |  |
| Blister | 0 | 1 (1.85%) | 1 (0.92%) | 0.21 |
| Pruritus | 0 | 3 (5.56%) | 3 (2.75%) | 0.06 |
| **Vascular disorders** |  |  |  |  |
| Hypertension | 3 (5.45%) | 0 | 3 (2.75%) | 0.06 |
| Phlebitis | 0 | 1 (1.85%) | 1 (0.92%) | 0.21 |
| **Investigations** |  |  |  |  |
| Alanine aminotransferase | 2 (3.64%) | 1 (1.85%) | 3 (2.75%) | 0.21 |
| Blood bilirubin abnormal | 0 | 4 (7.41%) | 4 (3.67%) | 0.07 |
| Blood bilirubin increased | 0 | 1 (1.85%) | 1 (0.92%) | 1.00 |
| Blood pressure systolic | 1 (1.82%) | 0 | 1 (0.92%) | 1.00 |
| Urine output decreased | 1 (1.82%) | 0 | 1 (0.92%) | 0.89 |
| Weight decreased | 2 (3.64%) | 1 (1.85%) | 3 (2.75%) | 0.41 |
|  |  |  |  |  |

NECT = nifutimox-eflonithine combination treatment. * indicates significant differences across treatment groups.
